# Adsorption-Based Mitigation of Azo Dye Toxicity: Removal of Direct Red 23 Using Amberlite XAD-4 Resin

**DOI:** 10.3390/toxics14060491

**Published:** 2026-06-04

**Authors:** Nicoleta Mirela Marin, Toma Galaon, Adriana Mariana Borș, Roxana Doina Trusca, Ludmila Motelica, Ovidiu Oprea

**Affiliations:** 1 National Research and Development Institute for Industrial Ecology ECOIND, Street Podu Dambovitei No. 57-73, District 6, 060652 Bucharest, Romania; tomagalaon@yahoo.com; 2 Department of Analytical and Physical Chemistry, University of Bucharest, 4-12 Regina Elisabeta Bd., 030018 Bucharest, Romania; 3 Department of Oxide Materials Science and Engineering, National University of Science and Technology Politehnica Bucharest, 1–7 Gh. Polizu, 060042 Bucharest, Romania; 4 National Institute for R&D for Optoelectronics-Subsidiary, Research Institute for Hydraulics and Pneumatics—INOE 2000-IHP, 040558 Bucharest, Romania; bors.ihp@fluidas.ro; 5 National Centre for Micro- and Nanomaterials, National University of Science and Technology Politehnica Bucharest, 313 Independence Boulevard, 060042 Bucharest, Romania; truscaroxana@yahoo.com (R.D.T.); ludmila.motelica@upb.ro (L.M.); ovidiu.oprea@upb.ro (O.O.); 6 Research Center for Advanced Materials, Products and Processes, National University of Science and Technology Politehnica Bucharest, Splaiul Independentei 313, 060042 Bucharest, Romania; 7 Academy of Romanian Scientists, 3 Ilfov St., 050045 Bucharest, Romania; 8 Faculty of Chemical Engineering and Biotechnologies, National University of Science and Technology Politehnica Bucharest, Gh. Polizu 1-7, 011061 Bucharest, Romania

**Keywords:** Amberlite XAD-4 resin, toxic Direct Red 23 dye, adsorption, isotherm, kinetics, reusability, SEM/EDX

## Abstract

The release of persistent azo dyes into aquatic systems remains a critical environmental and toxicological concern due to their high chemical stability, resistance to biodegradation, and potential to generate carcinogenic aromatic amines. This study evaluates the adsorption of Amberlite XAD-4 (X4), a hydrophobic polystyrene–divinylbenzene resin, for the removal of the toxic azo dye Direct Red 23 (DR 23) from aqueous solutions. Batch experiments were performed to assess the influence of contact time and initial dye concentration, supported by kinetic and equilibrium modeling. Adsorption proceeded through a multistage mechanism involving thin-layer diffusion, intraparticle diffusion, and final equilibrium, which was reached after 48 h. The pseudo-second-order kinetic model (PSO) with R^2^ = 0.9648 best described the adsorption behavior. Equilibrium data was fitted by the Langmuir isotherm (R^2^ = 0.9990), yielding a maximum adsorption capacity of 56.8 mg g^−1^, consistent with the experimentally observed saturation plateau. FTIR spectra revealed characteristic shifts in aromatic, –N=N– (≈1500 cm^−1^), and –SO_3_^2−^ (1180–1040 cm^−1^) bands, which, corroborating the data provided by SEM/EDX analysis, completes the adsorption of DR 23 on the X4 matrix. TG/DSC analysis showed modifications in thermal behavior after adsorption without compromising resin stability, supporting strong dye–resin interactions. Overall, the integrated kinetic, isotherm, spectroscopic, and thermal analyses demonstrate that X4 is stable and an adsorbent with desorption capability using chemical agents, highlighting its potential for mitigating the environmental and toxicological risks associated with azo dye contamination in wastewater.

## 1. Introduction

Today, the release of synthetic dyes into aquatic environments remains a major global environmental challenge due to their toxicity, persistence, and resistance to conventional biological degradation [1]. Industries such as textile, leather, paper, and dye manufacturing discharge large amounts of wastewater annually, with azo dyes representing ≈60–70% of all dyes used worldwide [2,3,4].

Azo dyes are characterized by one or more azo (–N=N–) linkages and complex aromatic structures that confer high stability but also recalcitrance to the environment. Among them, DR 23, a widely used anionic azo dye, poses significant risks to human health and ecology due to both its potential to form carcinogenic aromatic amines under reductive conditions and its strong color intensity, which inhibits photosynthesis in aquatic systems [5,6,7]. Consequently, the development of efficient and sustainable treatment technologies for the removal of DR 23 from wastewater is of critical importance.

Conventional wastewater treatment processes, including coagulation–flocculation, membrane filtration, advanced oxidation, and biological treatment, often fail to achieve complete removal of dye or are limited by issues such as high operational costs, sludge generation, or sensitivity to wastewater composition [8,9,10]. Adsorption has emerged as one of the most effective and versatile methods for dye removal due to its simplicity, cost effectiveness, high efficiency, and ability to treat a wide range of pollutants without generating harmful by-products [11]. Recent research has focused on developing advanced adsorbent materials with high surface area, chemical stability, and tunable surface functionalities to improve the adsorption performance of complex organic pollutants such as azo dyes [12].

The development of high-surface-area polymeric resins represents a significant advance in wastewater treatment. These materials, characterized by controlled porosity, high chemical stability, adaptable surface functionality, and well-defined polymeric networks, enable the efficient adsorption of persistent azo dyes. Their performance is based on mechanisms such as electrostatic interactions, hydrogen bonds, π–π interactions, and ion exchange processes [13].

Functionalization with groups such as amine, carboxyl, or hydroxyl further enhances selectivity and binding capacity. Furthermore, the incorporation of molecularly imprinted polymer structures provides highly specific recognition sites for the targeted removal of dyes [14].

Recent advances also include the development of biological and hybrid polymer systems, which offer more sustainable alternatives [15]. When designed as advanced composite systems, such as core–shell structures or inorganic hybrid polymers, these materials exhibit high adsorption capacity, rapid dye removal, and excellent regenerability over multiple cycles, and robust performance even in complex industrial effluents [16,17,18,19,20].

Polymeric adsorbents, especially microporous resins, have gained increasing attention due to their hydrophobicity, mechanical strength, and regeneration capability. Among these, X4, a non-ionic cross-linked polystyrene-divinylbenzene resin, has been widely studied for the removal of organic contaminants from aqueous solutions [21,22]. Its high surface area (~725–800 m^2^/g), uniform pore structure, and chemical stability make it suitable for adsorbing a broad range of hydrophobic and moderately polar compounds [23]. Specialized studies have demonstrated the effectiveness of X4 and its modified derivatives for removing dyes, pharmaceuticals, pesticides, and heavy metals from wastewater [14,24,25,26,27,28,29]. The adsorption capacity and selectivity towards specific pollutants are demonstrated by the functionalization of X4 with amines, chelating agents, or sulfonic acid groups, which has been shown to significantly improve these properties [13,30,31,32,33,34].

To the best of our knowledge, no previous studies have specifically investigated the adsorption of DR 23 onto X4. Existing literature reports XAD-type resins used for other dyes or organic pollutants [13,28,32,35,36,37,38,39], but no adsorption studies involving DR 23 and X4 were identified during our literature search. The main objective of this work was to evaluate the adsorption of the X4 for the removal of DR 23 from aqueous solutions. In addition, adsorption behavior was investigated through kinetic and isotherm modeling, while the solid matrices before and after adsorption were characterized using Fourier-transform infrared spectroscopy (FTIR), scanning electron microscopy (SEM), energy-dispersive X-ray spectroscopy (EDX), thermogravimetric and differential scanning calorimetric (TG/DSC) analyses, and desorption experiments.

## 2. Materials and Methods

### 2.1. Materials

X4 (Sigma-Aldrich (Merck, Darmstadt, Germany)) is a polymeric, non-ionic and hydrophobic adsorbent resin widely used for the separation and purification of organic compounds from aqueous streams or polar solvents. The resin is made up of a cross-linked polystyrene–divinylbenzene (PS-DVB) matrix, with a macroreticular structure that gives it excellent chemical and thermal stability. X4 has a specific surface area of about 750 m^2^/g, which allows for a high adsorption capacity and an average pore diameter of 100 Å, being used in a wide pH range (0–14), with good resistance to high temperatures (up to ~250 °C under certain drying conditions). The resin acts by physical adsorption (Van der Waals forces) and can be regenerated by washing with organic solvents (such as methanol or acetone) or by bases/acids, depending on the adsorbed substance’s nature [40].

DR 23 (Sigma-Aldrich (Merck, Darmstadt, Germany)) is a versatile, water-soluble synthetic azo dye, widely used in various industries due to its deep red color and ease of application, and it has a molecular weight of 813.72 g/mol [41].

The reagents used in the desorption studies—hydrochloric acid (HCl) 37% (used to prepare the 1 M HCl solution), ethanol (≥99.8%), methanol (≥99.8%), and 1 M sodium hydroxide (NaOH)—were purchased from Merck (Darmstadt, Germany).

### 2.2. Methods

#### 2.2.1. FTIR-ATR Analysis

FTIR spectra were obtained at room temperature using a Nicolet iS50R spectrometer (Thermo Fisher Scientific Inc., Madison, WI, USA), equipped with an Attenuated Total Reflectance (ATR) module. Samples were placed directly onto the ATR crystal to ensure adequate contact during measurement. For each sample, 32 scans were recorded over the spectral range of 4000–400 cm^−1^, with a spectral resolution of 4 cm^−1^.

#### 2.2.2. SEM/EDX Analysis

SEM and EDX analyses were carried out using a Quanta Inspect F50 field-emission scanning electron microscope (FEG-SEM, FEI, Hillsboro, OR, USA) with a spatial resolution of 1.2 nm. The microscope is equipped with an EDX detector, offering an energy resolution of 133 eV for the MnKα line, enabling detailed morphological and elemental characterization of the samples.

#### 2.2.3. TG-DSC Analyses

The thermal analysis TG-DSC for the resins and dye was performed with a Netzsch STA 449C Jupiter apparatus (NETZSCH-Gerätebau GmbH, Selb, Germany). The samples were placed in an alumina crucible, closed with a pierced lid, and heated with 10 K/min from room temperature up to 900 °C, under the flow of 50 mL/min dried air. An empty alumina crucible was used as the reference.

#### 2.2.4. Linearity of UV–Vis Method

**Figure 1:**
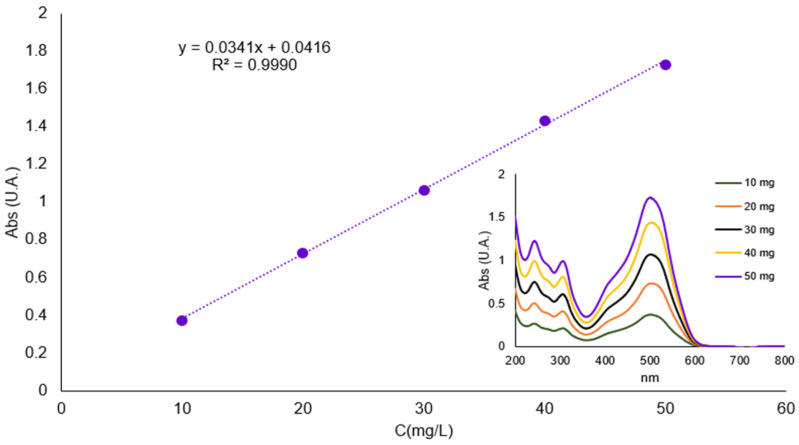
Graphical representation of A vs. C (mg/L) for linearity of the UV–Vis method. All reported values represent the mean of duplicate measurements, each showing a standard deviation below 3%.

The linearity of the UV–Vis method for the quantitative determination of DR 23 was evaluated by constructing a calibration curve in the concentration range of 10–50 mg/L. For this, a stock solution of 1 g/L DR 23 was prepared in distilled water and subsequently diluted to obtain 10, 20, 30, 40, and 50 mg/L. Initially, the maximum absorption wavelength (λ_max_) of DR 23 was determined by scanning the spectrum in the UV–Vis 200–800 nm region, and all absorbance measurements at λ_max_ = 500 nm using 1 cm quartz cuvettes were performed. Distilled water served as the blank. Each standard solution was analyzed under the same instrumental conditions, and the corresponding absorbance values were recorded as follows: 0.3731 (10 mg/L), 0.7289 (20 mg/L), 1.0645 (30 mg/L), 1.4322 (40 mg/L), and 1.7275 (50 mg/L). The calibration curve was constructed by plotting absorbance (A) vs. concentration (C, mg/L), resulting IN A = 0.0341C + 0.0416 (Figure 1).

The correlation coefficient (R^2^) was calculated to assess the fitting quality. The obtained R^2^ value confirmed good linearity of the UV–Vis method within the investigated concentration range, demonstrating compliance with the Beer–Lambert law under the selected analytical conditions. This linearity study validates the UV–Vis method as suitable for the quantitative determination of DR 23 in aqueous samples within the tested concentration interval.

### 2.3. Purification Procedure for X4

X4 was purified prior to use in order to remove residual monomers, organic impurities, and any adsorbed contaminants from manufacturing or storage. The X4 was first washed several times with distilled water to eliminate dust and suspended particles. After decantation, the X4 was treated with ethanol to remove organic residues, followed by repeated rinsing with distilled water until the washings became clear. X4 was then kept in 1 M HCl under stirring for 3 h to remove impurity of the matrix. After acid treatment, X4 was washed with distilled water until a neutral pH was reached. Finally, the purified X4 was dried and stored in a desiccator until use in adsorption experiments.

### 2.4. Procedure for Adsorption of DR 23 onto X4 at 25 °C Depending on Contact Time

Samples of 0.05 g X4 were weighed on an analytical balance and transferred into Erlenmeyer flasks. Then, 0.01 L of DR 23 solution with concentration of 300 mg/L was added to each sample. The mixtures obtained were stirred at different contact times that varied from 0.5 to 6, 12, 24, 36, 48, 50, and 62 h at 175 rpm (T = 25 ± 2 °C). At the end of each contact time, the mixture was filtered, and the obtained solutions were analyzed by UV–Vis spectrophotometry. The amount of DR 23 mass adsorbed at time t, (mg/g), was then calculated using Equations (1) and (2) [42,43].

(1) $$Q_{t}=\frac{\left(C_{i}-C_{t}\right)V}{m}$$

(2) $$R\left(\%\right)=\frac{C_{i}-C_{e}}{C_{i}}\times 100$$

where: C_i_ (mg/L) is the initial concentration of the DR 23 in the solution before adsorption at begins; C_t_ (mg/L) is the concentration of DR 23 at time during the adsorption process; C_e_ (mg/L) is the equilibrium concentration, measured after the system reaches adsorption equilibrium; V (L) the volume of the solution used in the experiment; m (g) is the X4 mass; Q_t_ (mg/g) is the adsorption capacity at time, t, representing how much DR 23 has been adsorbed per mass of X4; and R (%) is the removal efficiency, expressed as a percentage.

A control experiment was performed under the same conditions as presented previously, using a 300 mg/L DR 23 solution (0.01 L), maintained at 25 ± 2 °C and stirred at 175 rpm for contact times ranging from 0.5 to 62 h, but in the absence of the X4. UV–Vis analysis of the samples collected at each time point confirmed that the absorbance and concentration of DR 23 remained unchanged throughout the period, indicating that no dye degradation, photolysis, or spontaneous loss occurred under the experimental conditions applied.

### 2.5. Procedure for Adsorption of DR 23 onto X4 Depending on Initial Concentration

Samples of 0.05 g X4 were weighed on an analytical balance and transferred to Erlenmeyer flasks. Then, 0.01 L of DR 23 solutions with different initial concentrations of 50, 100, 150, 200, 250, 300, 350, and 400 mg/L were added to the X4. The obtained mixtures were stirred at 175 rpm at T = 25 ± 2 °C, at 48 h established as sufficient time to reach equilibrium. At the end of the contact time, the mixture was filtered, and the filtered solutions were analyzed by the UV–Vis method. The amount of DR 23 mass adsorbed at equilibrium (mg/g) was then calculated using Equation (3) [44,45].

(3) $$Q_{e}=\frac{\left(C_{i}-C_{e}\right)V}{m}$$

### 2.6. Regeneration of X4 Loaded with DR 23

For regeneration studies, 10 mL of 1 M NaOH, 1 M HCl, methanol (MeOH), 1 M HCl–MeOH (1:1 *v*/*v*), and 1 M NaOH–MeOH (1:1 *v*/*v*) were added, as well as over 0.1 g of X4 saturated resin with DR 23 (55 mg of DR 23/g of X4). The mixtures were stirred for 0.5 h at 175 rpm, T = 25 ± 2 °C. At the end of the stirring time, the mixture was filtered, and supernatant solutions were analyzed by the UV–Vis method.

## 3. Results

### 3.1. Kinetics of DR 23 Adsorption onto X4

**Figure 2:**
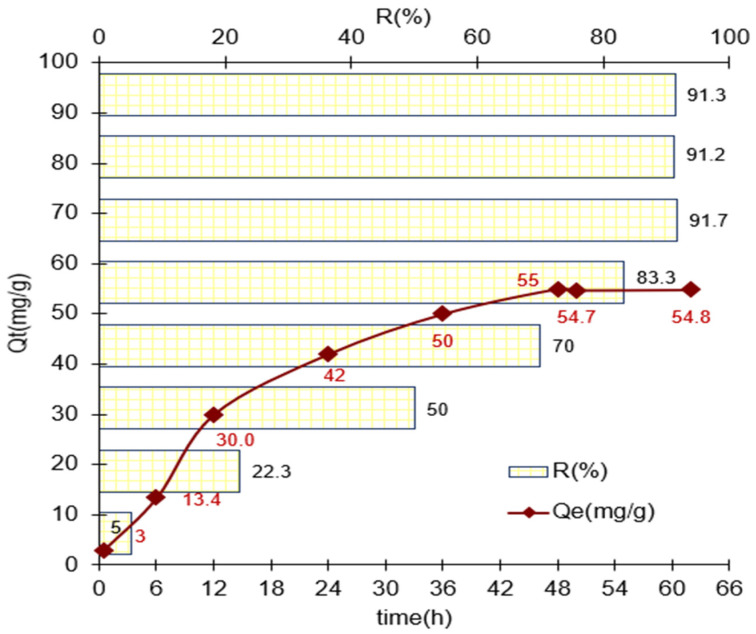
The influence of contact time regarding DR 23 onto X4 mass. All reported values represent the mean of duplicate measurements, each showing a standard deviation below 3%.

The kinetics of DR 23 adsorption onto X4 were evaluated at T = 25 ± 2 °C under constant agitation (175 rpm), using 0.05 g of X4 in 0.01 L of a 300 mg/L dye solution. The results obtained regarding the influence of contact time are presented in Figure 2.

It was observed that, during the first 30 min of contact, adsorption remained limited, with only 5% removal, and a Q_t_ value of 3 mg/g was obtained. As a result, in the initial phase, external resistance to mass transfer predominates, limiting the transport of DR 23 molecules from the bulk solution to the X4 surface. The initial diffusion rate is slow, consistent with the relatively large molecular size and complex aromatic structure of DR 23, which may inhibit rapid migration toward the resin pore entrances.

As the contact time increased to 6 h, the adsorption capacity reached 13.4 mg/g, while the removal efficiency was approximately 22%. This increase reflects the progressive reduction in boundary-layer resistance and the enhanced diffusion of dye molecules toward the external surface of the resin. Beyond this point, the transition from external mass-transfer control to intraparticle diffusion becomes evident.

Between 6 and 24 h, adsorption proceeded more rapidly, with the Q_t_ value increasing from 13.4 to 42 mg/g, and the R (%) registered an increase of ≈50% from 22% to 70%. The approximately linear increase in the Q_t_ value during this interval suggests that intraparticle diffusion becomes the predominant limiting step. The sustained driving force for mass transfer and the continuous availability of active sites in the polymer matrix characterize this active adsorption phase.

After 24 h, the adsorption rate was significantly reduced. At 36 h, Q_t_ reached 50 mg/g (83%), and at 48 h, it approached 55 mg/g (92%). The decrease in the adsorption rate reflects the progressive saturation of the adsorption sites and the decrease in the concentration gradients between the solution and the X4 surface. The adsorption capacity at 48 h is very close to the equilibrium capacity obtained from isotherm studies, indicating that the system has effectively reached equilibrium.

After 48 h, no significant changes were observed for the Q_t_ values, removal efficiency, or the equilibrium concentration. Between 50 and 62 h, the values of Q_t_ remained practically constant (54.7–54.8 mg/g), and R (%) was stabilized at ≈91%, with C_e_ around of 26 mg/L. This stability confirms that the adsorption process has reached equilibrium.

So, the kinetic profile of DR 23 adsorption on X4, taking into account the results obtained regarding the influence of contact time, is characterized by: (i) a slow initial phase, dominated by resistance to external mass transfer; (ii) a prolonged intraparticle diffusion phase, during which most of the adsorption takes place; and (iii) a final equilibrium phase, in which the adsorption sites become saturated and the adsorption slows down (Figure 2).

#### Kinetic Models

The kinetic evaluation of DR 23 adsorption onto X4 shows that the process evolves gradually toward equilibrium, with Q_t_ (mg/g) stabilizing after approximately 48 h. This slow approach confirms that both film diffusion and intraparticle diffusion contribute to the overall rate of adsorption. The final equilibrium capacity (~55 mg/g) is consistent with the isotherm results, demonstrating the reliability of the adsorption behavior under the applied experimental conditions. These findings underline the strong affinity of X4 for DR 23 and the importance of extended contact time, particularly for bulky azo dyes with complex aromatic structures.

The kinetic parameters corresponding to the Weber–Morris (intraparticle diffusion), Elovich, pseudo-first-order (PFO), and pseudo-second-order (PSO) models were determined by linear regression using Equations (4)–(7) and the resulting values are presented in Table 1 [46,47,48].

k_id_ is the rate at which dye molecules are removed into the adsorbent’s internal pores. Higher k_id_ (mg/g·h^−0.5^) values indicate faster intraparticle transport. C corresponds to the thickness of the boundary layer surrounding the X4 particles; if C ≠ 0, this suggests that intraparticle diffusion is not the only rate-controlling mechanism. The constant α denotes the initial adsorption rate, reflecting rapid adsorption that begins on the heterogeneous surface. Parameter β is associated with the extent of surface coverage and the activation energy required for adsorption; also, it describes the decrease in the adsorption rate as the surface becomes progressively occupied. k_1_, k_2_: rate constant of the PFO and PSO. Q_e_: calculated adsorption capacity at equilibrium (amount adsorbed per gram of X4 at equilibrium).

The Weber–Morris plot shows a linear relationship but does not pass through the origin, as indicated by the intercept C = 1.11 mg/g, demonstrating that intraparticle diffusion is involved but is not the sole rate-limiting step. The correlation coefficient (R^2^ = 0.9607) supports a multistage adsorption mechanism: rapid film diffusion at the external surface followed by the gradual penetration of DR 23 molecules into the internal pores of the X4. The non-zero intercept confirms the presence of boundary-layer resistance during the initial adsorption stage.

The experimental data were also fitted using the Elovich kinetic model, yielding a good correlation (R^2^ = 0.9550). The calculated constants (α = 0.002 mg/g·h and β = 0.0711 g/mg) reflect a decreasing adsorption rate over time, consistent with the progressive occupation of high-energy sites and the subsequent slower adsorption on lower-energy regions.

The PFO model provides the weakest fit (R^2^ = 0.8970), indicating that it does not adequately describe the adsorption kinetics of DR 23 on X4. The calculated equilibrium capacity (Q_e_ = 85.07 mg/g) overestimates the experimental value, and the rate constant (k_1_ = 0.11 g/mg·h) does not capture the diffusion-controlled nature of the process. These discrepancies confirm that the adsorption rate is not governed by first-order physisorption.

**Figure 3:**
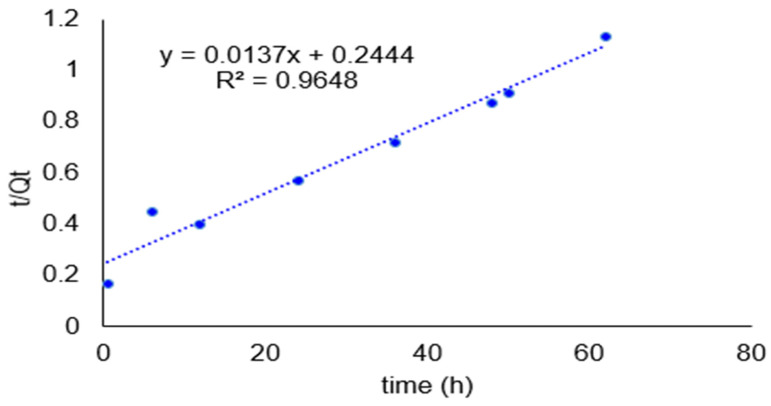
Linearized plot of PSO regarding DR 23 adsorption on X4.

The PSO model provides the best description of the kinetic data, with the highest correlation coefficient (R^2^ = 0.9648) (Figure 3). The rate constant k_2_ = 0.001 g/mg·h and the calculated Q_e_ = 73 mg/g suggest that adsorption occurs primarily through physisorption, driven by donor–acceptor interactions between the electron-rich azo/aromatic groups of DR 23 and the electron-deficient aromatic rings of the X4 resin [49]. Although the calculated Q_e_ value slightly underestimates the experimental value, such deviations are typical in systems where diffusion limitations influence the final stages of adsorption.

The Q_e_ value predicted by the PSO model (73 mg/g) is higher than the experimentally determined equilibrium capacity (≈55 mg/g). Such deviations are frequently reported in adsorption systems where intraparticle diffusion significantly contributes to the overall rate. The PSO model assumes that adsorption is controlled exclusively by surface interactions and does not fully incorporate the diffusion limitations that become dominant in the later stages of uptake, particularly for bulky azo dye molecules such as DR 23. As equilibrium is approached, the progressive saturation of internal pores and the decreasing concentration gradient slow the actual adsorption rate, resulting in an experimental Q_e_ lower than the theoretical value. Thus, the overestimation provided by the PSO model reflects the combined influence of diffusion resistance and pore-filling constraints rather than a deficiency in the kinetic description. Correlation-based model selection confirms the PSO model as the most appropriate kinetic representation for DR 23 adsorption onto X4, followed by the intraparticle diffusion and Elovich models. The overall analysis indicates a multistep adsorption mechanism involving rapid external mass transfer, slower intraparticle diffusion, and final saturation of the internal porous structure, consistent with physisorption on a heterogeneous surface and with the structural complexity of azo dyes and the porous architecture of the X4.

### 3.2. Adsorption Behavior of DR 23 on the X4 Adsorbent

**Figure 4:**
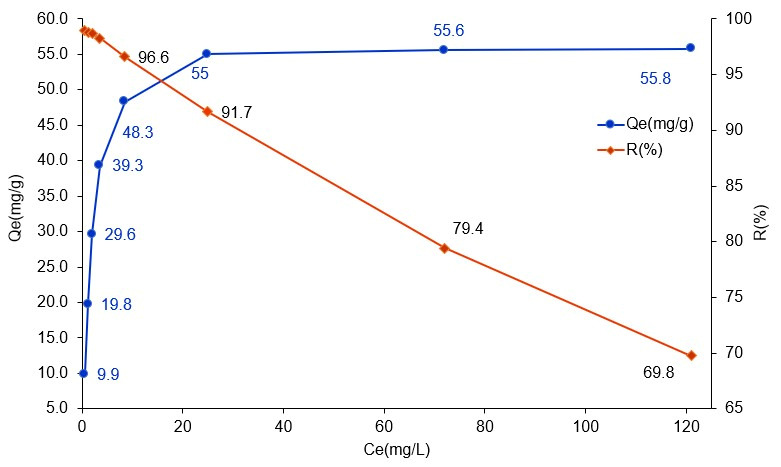
Adsorption of DR 23 onto X4 mass in function of initial concentration. All reported values represent the mean of duplicate measurements, each showing a standard deviation below 3%.

The adsorption behavior of DR 23 on the X4 was investigated under controlled batch conditions (T = 25 ± 2 °C, 175 rpm, 0.05 g X4 in 0.01 L of solution and a contact time of 48 h). The experimental results presented in Figure 4 reveal distinct trends regarding adsorption capacity and removal efficiency as a function of the initial dye concentration, which provides valuable information about the interaction mechanisms between DR 23 molecules and the hydrophobic polymer matrix of X4.

The adsorption capacity increased significantly with the initial dye concentration increase that varied from 9.9 mg/g at 50 mg/L to ≈55.0 mg/g at 300 mg/L. This continuous increase reflects the intensification of the driving force for mass transfer at higher dye concentrations, which support the diffusion of DR 23 molecules from solution to the X4 mass. The plateau observed at 300 mg/L indicates that the available adsorption sites are approaching saturation. The behavior of Q_e_ at the plateau suggests a maximum adsorption capacity at ≈56 mg/g for DR 23.

In contrast to the increasing value of Q_e_, removal efficiency showed a decreasing trend as the initial dye concentration increased. At low concentrations (50–150 mg/L), removal efficiencies were remarkably high (98.7–99%), demonstrating the strong affinity of DR 23 for the aromatic, nonionic surface of X4. This behavior is in line with the hydrophobic and π-electron-rich structure of X4, which facilitates π–π interactions with the aromatic rings of DR 23.

When the initial concentration exceeded 200 mg/L, the removal efficiency gradually decreased, reaching 69.8% (400 mg/L). This decrease reflects the progressive saturation of the adsorption sites: once a substantial portion of the X4 surface is occupied, additional dye molecules remain in solution, leading to higher equilibrium concentrations (C_e_). The decrease in removal efficiency thus provides a direct measure of the finite number of active adsorption sites on X4.

The equilibrium concentration increased from 0.5 mg/L at an initial concentration of 50 mg/L to 121 mg/L at 400 mg/L, confirming that X4 becomes increasingly saturated at higher dye concentrations. The rapid increase in C_e_ (mg/L) above 250 mg/L corresponds to the plateau observed in Qe and the decrease in removal efficiency, supporting the conclusion that the adsorption process is limited by the number of available active sites.

Overall, the experimental data demonstrates that X4 exhibits a strong adsorption affinity for DR 23, at low to moderate concentrations, where nearly complete removal is achieved. The saturation behavior observed at higher concentrations suggests that the adsorption mechanism is dominated by the formation of a monomolecular layer on a finite number of homogeneous sites, consistent with the Langmuir isotherm assumptions.

#### Isotherm Models for Adsorption Studies of DR 23 onto X4 Mass

The equilibrium adsorption data for DR 23 onto X4 was analyzed using the Langmuir (Equation (8)), Freundlich (Equation (9)), Temkin (Equations (10) and (11)), and Dubinin–Radushkevich (D–R) (Equations (12)–(14)) isotherm models to elucidate the adsorption mechanism and the physicochemical characteristics of the resin surface (Table 2) [50,51,52,53,54,55]. The experimental equilibrium capacities increased with increasing initial dye concentration until reaching a plateau at ≈55–56 mg/g, indicating saturation of the available adsorption sites.

(8) $$\frac{C_{e}}{Q_{e}}=\frac{1}{bQ_{m}}+\frac{C_{e}}{Q_{0}}$$

(9) $$\ln Q_{e\;}=\ln K_{f}\;+\frac{1}{n}{\mathrm{lnC}}_{e}$$

(10) $$Q_{e}=\frac{\mathrm{RT}}{b_{T}}\ln\;(AC_{e})$$

and can be linearized as follows:

(11) $$Q_{e}=\;\mathrm{BlnA}\;+\;\mathrm{Bln}C_{e}$$

(12) $$\ln Q_{e}=\ln q_{m}-\beta \varepsilon^{2}$$

(13) $$\varepsilon =\mathrm{RTln}(1+\frac{1}{C_{e}})$$

(14) $$E=\frac{1}{\sqrt{2\beta }}$$

where: $Q_{m}$ (mg/g) denotes the maximum sorption capacity of the X4. The parameter b (L/mg) represents the Langmuir constant, which characterizes the affinity between the X4 and the DR 23. The dimensionless separation factor $R_{L}$ is employed to assess the favorability of the sorption process. The correlation coefficient $R^{2}$ indicates the goodness of fit of the applied isotherm model. The Freundlich constant $K_{f}$ (mg/g) reflects the sorption capacity of the X4, while the exponent n describes the sorption intensity and surface heterogeneity. The Temkin constant $b_{T}$ (J/mol) is associated with the heat of sorption. Parameter $q_{m}$ (mg/g), derived from the Dubinin–Radushkevich model, represents the theoretical monolayer adsorption capacity, and E (kJ/mol) corresponds to the mean sorption energy of adsorption.

**Figure 5:**
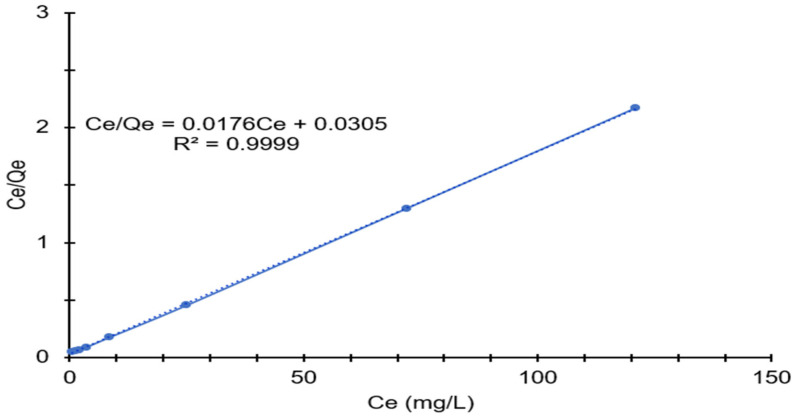
Linearized form of Langmuir isotherm.

The Langmuir isotherm was evaluated using its linearized form (Equation (8)), where the ratio C_e_/Q_e_ is plotted against C_e_, allowing for the determination of both the maximum adsorption capacity Q_m_ and the Langmuir constant b (Figure 5). The Langmuir model provided the best fit to the experimental data (R^2^ = 0.9990), demonstrating that the adsorption of DR 23 onto X4 follows a monolayer adsorption mechanism. The calculated maximum monolayer capacity (Q_m_ = 56.8 mg/g) is consistent with the observed plateau in the experimental data, confirming that the X4 possesses a well-defined number of active sites. The Langmuir affinity constant (b = 0.58 L/mg) indicates a favorable interaction between DR 23 and the X4 surface.

The Freundlich isotherm parameters were obtained by applying the logarithmic form of the model (Equation (9)), in which ln Q_e_ is linearly correlated with ln C_e_. This approach enables the estimation of the Freundlich constant K_f_ and the sorption intensity n. The Freundlich model also described the data reasonably well (R^2^ = 0.7675), indicating that surface heterogeneity contributes to the adsorption process. The Freundlich constant (K_F_ = 13.6) reflects a high sorption capacity at low equilibrium concentrations, while the heterogeneity factor (n = 3.63) confirms that adsorption is favorable. The simultaneous applicability of both Langmuir and Freundlich models suggests that X4 contains adsorption sites with different binding energies.

The Temkin isotherm was analyzed by expressing the relationship between Q_e_ and C_e_ in its linearized form, Q_e_ = B ln A + B ln C_e_ (Equation (11)), which permits the calculation of the Temkin constants A and B, and subsequently the heat-related parameter b_T_. The Temkin model yielded a moderate correlation (R^2^ = 0.8853), indicating that the heat of adsorption decreases linearly with increasing surface coverage. The Temkin constant (B = 8.45) suggests that the initial adsorption of DR 23 occurs on higher-energy sites, followed by progressively lower-energy sites. The Temkin heat parameter (b_T_ = 288 J/mol) is characteristic of weak, non-specific interactions, consistent with physical adsorption.

The Dubinin–Radushkevich (D–R) model was applied by plotting ln Qe against the square of the Polanyi potential ε, defined as ε = RT ln (1 + 1/C_e_) (Equation (13)). From the slope of the linearized D–R equation, the constant β was obtained, which was further used to calculate the mean sorption energy E = 1/√(2β) (Equation (14)) and the theoretical monolayer capacity q_m_. The D–R isotherm has provided additional insight into the adsorption mechanism.

The mean adsorption energy (E = 1.58 kJ/mol) is significantly below the threshold typically associated with chemisorption, confirming that the adsorption of DR 23 onto X4 is governed by physisorption. The D–R monolayer capacity (q_m_ = 47.1 mg/g) closely matches the Langmuir value, reinforcing the reliability of the estimated adsorption capacity.

The combined isotherm analysis reveals that the adsorption of DR 23 onto X4 is a favorable, predominantly physical process, characterized by monolayer adsorption on a heterogeneous surface. The combination of moderate adsorption capacity, low adsorption energy, and favorable equilibrium parameters suggests that X4 is an effective and regenerable adsorbent for the removal of azo dyes from aqueous solutions.

### 3.3. Desorption Studies

**Figure 6:**
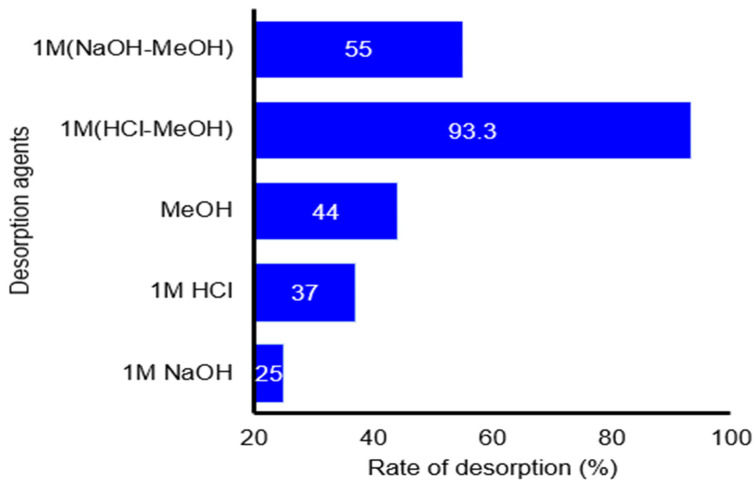
Regeneration of X4 loaded with DR 23 using different chemical agents. All reported values represent the mean of duplicate measurements, each showing a standard deviation below 3%.

The desorption behavior of DR 23 from the X4 polymeric resin was evaluated using 1 M NaOH, 1 M HCl, MeOH, 1 M (NaOH–MeOH), and 1 M (HCl–MeOH) as desorption agents. The results reveal significant differences in desorption efficiency depending on the chemical nature of the eluent. Experimental data obtained at desorption studies is presented in Figure 6.

Recent and relevant studies have extensively examined the adsorption and desorption behavior of azo dyes—particularly DR 23—as well as the regeneration efficiency of polymeric resins, as reported in the literature [9,11,56,57,58,59,60,61,62].

Analyzing the experimental results, the lowest desorption efficiency was obtained with 1 M NaOH, which achieved only 25% desorption. This suggests that alkaline conditions are not favorable for breaking the interactions between DR 23 and the hydrophobic matrix of X4. The DR 23 molecule, being an anionic azo dye, is less likely to be repelled by hydroxide ions, and the resin–dye interactions remain largely intact.

Moderate improvement was observed when 1 M HCl was used, resulting in 37% desorption. Acidic conditions may partially protonate functional groups on the dye, reducing its affinity for the resin. However, efficiency remains limited, indicating that protonation alone is insufficient to fully disrupt the adsorption forces.

A more pronounced effect was obtained with methanol (MeOH), which produced 44% desorption. As an organic solvent, methanol can weaken hydrophobic interactions between the dye and the resin matrix. This suggests that DR 23 is held on X4 primarily through non-polar interactions, which methanol can partially overcome.

The most significant enhancement in desorption efficiency was observed when methanol was combined with acid or base solutions. The mixture 1 M HCl–MeOH achieved an exceptionally high desorption rate of 93.3%, indicating a synergistic effect. In this case, methanol disrupts hydrophobic interactions, while HCl protonates dye molecules, reducing their affinity for the resin. Together, these mechanisms almost completely release the dye from the polymer matrix.

Similarly, the mixture 1 M NaOH–MeOH resulted in 55% desorption, higher than either NaOH or methanol alone. Although the synergy is less pronounced than in the acidic mixture, the combination still enhances desorption by simultaneously weakening hydrophobic interactions and altering the dye’s ionization state.

The results clearly demonstrate that desorption of DR 23 from X4 is strongly influenced by the chemical environment created by the desorbing agent. Hydrophobic interactions appear to play a major role in dye retention, as indicated by the improved performance of methanol-containing eluents. Additionally, the dramatic increase in desorption efficiency with the HCl–MeOH mixture suggests that protonation of the dye, combined with solvent effects, is the most effective strategy for complete dye recovery.

For regeneration of X4 in dye-removal processes, the HCl–MeOH mixture stands out as the optimal desorbing agent, providing nearly complete desorption within a short contact time. This ensures efficient resin reuse and reduces operational costs. The findings also highlight the importance of selecting eluents that simultaneously target both hydrophobic and ionic interactions for maximum desorption efficiency.

### 3.4. FTIR-ATR Spectra of Solid Samples

#### 3.4.1. Characteristic Signals for X4 Pure Resin

**Figure 7:**
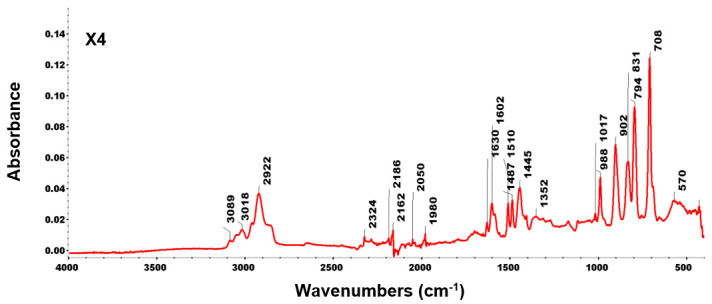
FTIR spectra of X4.

The main signals identified in the FTIR spectrum of X4 (Figure 7) are as follows: The region 3200–3550 cm^−1^/3400 cm^−1^ has a wide band as a result of the water adsorbed into the porous structure of the resin or the presence of groups –OH. The region 3000–3100 cm^−1^/3018.27–3078 corresponds to stretching vibrations of the aromatic C–H bonds. The region 2100–1620 cm^−1^/2050–1601.80 includes low-intensity bands (overtones) typical for the aromatic ring substitution mode. Region 2850–2950 cm^−1^: stretching vibrations of aliphatic C–H bonds (from ethylene bridges and methylene groups of the polymer backbone). A strong band appears at 2921.79 cm^−1^.

At 1601.80, 1488.67 and 1445.44 cm^−1^, intense bands corresponding to the stretching vibrations of the C=C bonds in the aromatic core are observed. In region 708.25–902.45 cm^−1^, out-of-plane bending vibrations for C–H bonds in benzene rings are observed. The range of ≈708.27 cm^−1^ and ≈794.27 cm^−1^ suggests the presence of monosubstituted (styrene) benzene rings. ~831.18 cm^−1^: characteristic of para-disubstituted benzene rings (divinylbenzene) and typical of unmodified polystyrene–divinylbenzene matrices [63]. The spectrum shows lines at 1500 cm^−1^ and 1355 cm^−1^/1510.28–1352.46 (indicating the presence of NO_2_ groups), which suggests chemical modifications in the resin (functionalization). The lack of bends in the 1700 cm^−1^ region indicates the lack of C=O groups (oxidation or adsorption of organic compounds).

#### 3.4.2. Characteristics Signals for DR 23

FTIR analysis allows for the identification of specific functional groups in its complex structure as a dye with sulfonic and urea groups [64].

**Figure 8:**
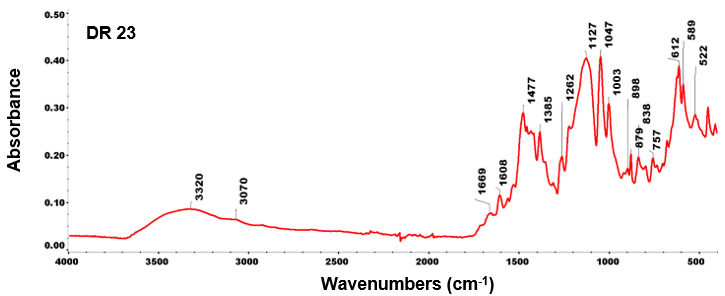
FTIR spectra of DR 23.

The FTIR spectrum of the DR 23 is shown in Figure 8. The characteristic bands are presented below.

Region 3200–3550 cm^−1^/3320.07 cm^−1^ is characterized by O–H or N–H stretching vibrations, a wide and intense band corresponding to the stretching vibrations of the –OH (hydroxyl) and -NH (amino/urea) groups. The width of the band is caused by the presence of inter- and intramolecular hydrogen bonds.

Region 1600–1650 cm^−1^/1607.93 cm^−1^ is characterized by carbonyl group stretching vibrations (C=O) from the urea bridge of the dye and may also include vibrations of the C=N bond in the aromatic system.

Region 1450–1650 cm^−1^/1476, 83–1607.93 cm^−1^ corresponds to the azo and aromatic system. Vibrations are recorded—multiple peaks between 1250–1400 cm^−1^ and 1262.09–1384.55 cm^−1^ that are often attributed to the stretching vibration of the azo group (–N=N–), although this may be weak or overlapping but can be associated with aromatic skeleton vibrations (benzene and naphthalene rings) [65].

Region 1000–1200 cm^−1^/1002.90–1127.49 cm^−1^ corresponds to the sulfonic groups; intense drops with values of 112.49 cm^−1^ and 1047.24 cm^−1^ appear that correspond to the asymmetrical and symmetrical stretching vibrations of the S=O bond in the sulfonate groups (–SO_3_Na) that give the solubility of the dye in water.

Region 450–1005 cm^−1^/451.69–1002.90 cm^−1^ corresponds to the out-of-plane deformation vibrations of the aromatic C–H bonds, specific to the substitution mode on the benzene and naphthalene rings; the disappearance or displacement of these droplets indicates the breakdown of the molecular structure of the dye [66].

#### 3.4.3. X4 Loaded with DR 23

**Figure 9:**
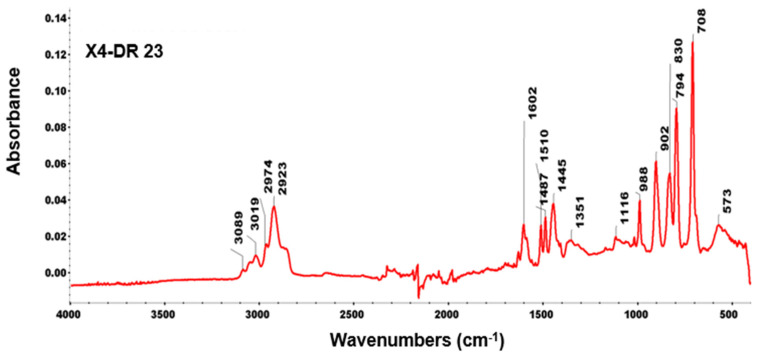
FTIR spectra of X4 loaded with DR 23.

The characteristic absorption bands of the X4 loaded with the DR 23 identified in Figure 9 highlight the structural changes that occur by the adsorption or chemical binding of the dye on the polystyrene–divinylbenzene polymer matrix, as follows:

Region 1520–1450 cm^−1^/1445.44 cm^−1^, 1486.67 cm^−1^, 1510.48 cm^−1^ corresponds to the azo groups -N=N-, which confirms the presence of azo bonds specific to the dye; this confirms the fixation of the dye on the resin. The azo dye contains –N=N–, –SO_3_^−^ groups and aromatic nuclei.

Region 1150–900 cm^−1^ includes a series of sulfonic –SO_3_^−^ groups of the adsorbed dye, which present intense bands in the range of 902.45–115.64 cm^−1^, representing symmetrical and asymmetrical stretching vibrations of the S=O groups.

Region 2900–3200 cm^−1^/2922.66–3019.54 cm^−1^ corresponds to a wide band indicating the presence of hydroxyl (–OH) groups in the dye structure, but also possible hydrogen bonds formed between the dye and the resin surface.

After the adsorption of the dye, new bands or displacements of the existing ones appear, which confirm the presence of the functional groups of DR 23, suggesting π–π interactions between the aromatic nuclei, hydrophobic interactions, possibly weak electrostatic interactions [67]. There is an intensification of the aromatic bands in the region of 1620–1350 cm^−1^/1351.11–1601.80 cm^−1^ due to the superimposition of the aromatic nuclei of the dye over those of the resin.

Based on the spectrum, we can deduce that adsorption is mainly controlled by:

(i) π–π stacking interactions between the aromatic nuclei of the resin and the dye [68];
(ii) Hydrophobic interactions due to the non-polar character of the polymer matrix [69];
(iii) Possible weak electrostatic interactions between the sulfonate groups of the dye and the surface of the adsorbent [70].

Therefore, FTIR confirms the adsorption of DR 23 on X4 by:

(1) The appearance of the characteristic bands –N=N– and –SO_3_^−^;
(2) Increasing the intensity of aromatic bands in the region 1620–1350 cm^−1^;
(3) Small displacements of the polymer strips [71].

These changes indicate that the dominant mechanism is the π–π interaction between the aromatic structures of the dye and resin [67].

After the adsorption process of DR 23, the FTIR spectrum of the charged resin shows significant changes. The appearance or intensification of the bands in the range of 1520–1450 cm^−1^ is observed, attributed to the characteristic vibration of the azo group (–N=N–), as well as of the bands in the ranges of 1150–900 cm^−1^ and 2900–3200 cm^−1^, corresponding to vibrations S=O and S–O of the sulfonate groups (–SO_3_^−^) present in the dye structure. Also, a slight increase in the intensity of the aromatic bands is observed in the region of 1620–1350 cm^−1^, suggesting the overlap of vibrations coming from the aromatic nuclei of the dye and the polymer matrix [72].

These spectral changes confirm the adsorption of the dye on the surface of the resin and indicate that the process is dominated by π–π interactions between the aromatic nuclei of the dye and the aromatic structure of the resin, as well as hydrophobic interactions [67].

The comparative analysis of the spectra of X4 before adsorption, DR 23 and X4 after dye adsorption allows for the identification of the mechanisms involved [72].

The analysis focuses on band displacements, intensity variations and the appearance/disappearance of characteristic vibrations.

FTIR analysis confirms that the adsorption of DR 23 on X4 leads to changes in the characteristic bands of the system, reflected by frequency shifts, intensity variations and the emergence of new bands. These changes indicate that the adsorption mechanism is dominated by π–π and hydrophobic interactions, specific to adsorption on aromatic polymer resins [69,70], also evaluated through kinetic and microstructural modeling frameworks (15), complemented by advanced morphological surface mappings [73,74].

The interpretation was made by comparing the FTIR spectra of the adsorbent before adsorption, of the dye and of the adsorbent after the adsorption process.

In the area of 1000–1200 cm^−1^, there is a band associated with the stretching vibrations of the C–N bonds. The presence of these bands confirms the anionic character of the DR 23.

After the adsorption process of DR 23 on the surface of X4, several changes in the FTIR spectrum are observed. The band characteristic of aromatic C=C vibrations, initially located at about 1601 cm^−1^ in the spectrum of the adsorbent, shows a slight shift towards lower values of the wavenumber of 1510,48 cm^−1^ and an increase in intensity, suggesting interactions between the aromatic nuclei of the adsorbent and those of the dye. Also, in the adsorbent spectrum, after adsorption, bands from the region of 1150–900 cm^−1^ appear or become more pronounced, corresponding to the S=O vibrations of the sulfonate groups of the dye. This observation confirms the presence of DR 23 molecules on the surface of the adsorbent. At the same time, the bands corresponding to the aromatic C–H vibrations in the range of 3200–3550 cm^−1^ show variations in intensity, suggesting changes in the chemical environment of the aromatic nuclei of the resin after the adsorption of the dye.

#### 3.4.4. Interpretation of the Adsorption Mechanism

The changes observed in the FTIR spectra indicate that the adsorption process of DR 23 on X4 is dominated by π–π interactions between the aromatic nuclei of the polymer resin and the extensive aromatic system of the dye. In addition, the presence of sulfonate groups in the dye molecule can favor weak electrostatic interactions or hydrogen bonds with the adsorbent surface. Thus, the FTIR analysis confirms that the adsorption of the dye on X4 mainly involves physical interactions of type π–π and hydrophobic interactions, without major structural changes in the adsorbent and, therefore, the analysis focuses on band displacements, intensity variations and the appearance/disappearance of vibrations characteristic of the spectral changes occurring after adsorption and their correlation with the mechanism of the interaction. The comparative analysis of the spectra of X4 before adsorption, DR 23 and X4 after dye adsorption allows for the identification of the mechanisms involved.

FTIR analysis confirmed that DR 23 was adsorbed onto X4. Adsorption occurs mainly through hydrogen bonds (–OH with –SO_3_^−^/–NH), electrostatic interactions, and π–π stacking between the dye molecules and the polymer surface. The structural integrity of X4 remains intact, indicating its stability and reusability. These results provide clear information about the adsorption mechanism and explain the high affinity of the X4 adsorbent for anionic dyes. Adsorption isotherms indicate both single-layer and multilayer coating, reflecting the presence of heterogeneous binding sites, while kinetic studies follow the pseudo-second-order model, suggesting that functional group interactions control the rate of adsorption. These results demonstrate that X4 exhibits a high capacity for DR 23 removal, being an effective and reusable adsorbent for anionic dyes.

### 3.5. SEM and EDX Characterization of X4 and X4 Loaded with DR 23

#### 3.5.1. Morphological Analysis of X4 Resin (SEM)

**Figure 10:**
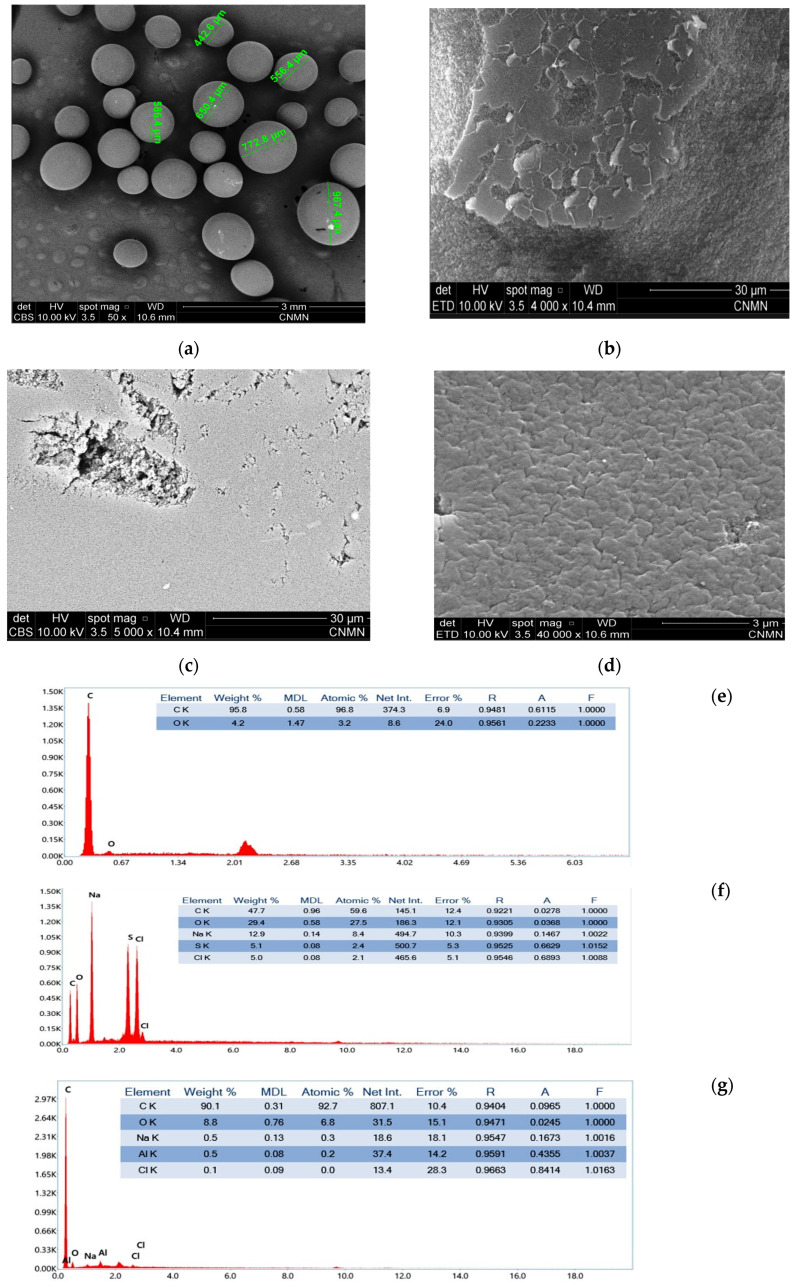
SEM image of (**a**) X4 50×, (**b**) X4-DR 23 4000×, (**c**) X4-DR 23 5000×, (**d**) X4-DR 23 40,000×, (**e**) EDX for X4, (**f**) EDX for DR 23 and (**g**) EDX for X4-DR 23.

An SEM image at 50× magnification provides an overview of the overall morphology of X4, complementing the structural details of the resin particles (Figure 10a). These are spherical beads, typical of suspension polymerization, intact, without major cracks or fragmentation, indicating good mechanical strength of the styrene–divinylbenzene matrix [63]. This shape ensures optimal flow in the adsorption columns and a uniform contact surface. The image shows a mixture of different sizes (from ~440 µm to ~750 µm), and this polydispersity indicates a normal distribution for the commercial X4 (which is not monodisperse). The particle size (e.g., 440 µm, 558 µm, 722 µm) confirms that the resin falls within the standard specifications (typically between 300 and 1200 µm for X4). This image shows that the resin retained its physical shape after contact with the dye solution, confirming that X4 is a stable support that does not chemically degrade in the presence of DR 23.

The structure of X4 visible on the right and bottom of the image shows the granular, porous and slightly irregular texture of the matrix at 4000× magnification (Figure 10b).

The left central area shows a crust or fragmented layer covering the resin surface. These “islands” of material represent DR 23 molecules that have clumped together and fixed themselves onto the active sites of the resin [64]. The mosaic or crusty (dry) appearance of the dye layer suggests that the adsorption was not just molecular (thin layer), but a consistent layer was formed, which, upon drying the sample for SEM, contracted and cracked. The increase in oxygen and decrease in carbon compared to pure resin indicates the presence of this visible layer that masks the chemical composition of the polymer support. The elements Na and Cl (traces) are trapped in the crystal structure of the dye layer in the image. Visualization of these massive deposits indicates the adsorption of DR 23 onto X4 [67]. At the 30 mm scale, it can be seen that the dye tends to saturate the surface, forming a visible layer of adsorbent.

The SEM image, taken at 5000× magnification, highlights the heterogeneous nature of the surface after adsorption, confirming that the active sites are unevenly/non-uniformly distributed (Figure 10c) [68]. The porous structure provides a clear view of deep cavities and irregularities in the polymer matrix [71]. The macroreticular X4 allows large DR 23 molecules to penetrate through these pits/dimples inside the pearl [66]. In the upper left, an area heavily loaded with material (the adsorbed dye) is observed that appears to completely fill the natural roughness of the resin. The dye does not just deposit as a monomolecular layer, but tends to form aggregates in areas of high surface energy (cracks and pores) [75], such as the large deposit on the left (Figure 10c). The very irregular texture and the presence of small, very bright dots (backscattered electrons) could suggest the presence of mineral micro-inclusions (e.g., aluminum oxide 0.5 wt% elemental analysis). The rough material is loaded with sulfonate groups of the dye [65]; so, the appearance of the pores is modified as a result of dye deposition.

#### 3.5.2. High-Magnification Evaluation of Dye Aggregates

The image presented in Figure 10d taken at an extreme magnification of 40,000× shows a surface texture of the X4-DR23 system, a fusion of pearls [69]. The surface is not smooth, but has a very fine granular texture. This is a direct result of the deposition of DR 23 on the polymer matrix [66]. Small grooves or demarcation lines can be observed between the dye aggregates, an appearance that appeared during the drying process of the sample for SEM analysis, which indicates the thickness of the adsorbate layer formed by surface forces. The density of these nodular formations suggests a high occupancy of the active sites on the external surface of the resin, which explains the increase in the percentage of oxygen (8.8%) and the presence of Na (0.5 wt%). The size of the surface aggregates is in the order of nanometers, which demonstrates the efficiency of X4 in retaining complex DR 23 molecules [76]. To conclude, SEM images of X4 reveal a granular morphology, with irregular, well-defined particles and a relatively smooth texture. The surfaces are dominated by open macro- and mesopores, visible as uniform depressions, confirming the resin’s high porosity and internal accessibility. This structure is favorable for adsorption processes, as it allows for the rapid diffusion of molecules into the porous network. After contact with DR 23, the morphology changes significantly: the surface becomes much rougher, with agglomerations and continuous deposits distributed across the particles. At the same time, the particle contours are less defined, a sign of coverage with an organic layer. The visible pores of X4 are partially or completely blocked, indicating the penetration of DR 23 into the porous network. These changes confirm the substantial adsorption of DR23 on the surface and within the X4 material, both through physical π–π stacking interactions and via van der Waals forces.

#### 3.5.3. Elemental Composition and EDX Microanalysis

Comparative EDX analysis reveals clear changes in the elemental composition of X4 following DR 23 adsorption (Figure 10e–g) [69]. The resin before adsorption has a strongly carbonic structure, dominated by C (95.8 wt%) and a small amount of O (4.2 wt%), with no detectable inorganic elements, confirming the purity of the material. In contrast, DR 23 exhibits a distinct chemical fingerprint, characterized by the presence of Na (12.9 wt%), S (5.1 wt%), and Cl (5.0 wt%), associated with sulfonate groups and its commercial form; wt% refers to the weight fraction of each detected element expressed as a percentage.

After adsorption, X4-DR 23 shows a decrease in carbon to 90.1 wt% and an increase in oxygen to 8.8 wt%, indicating the presence of sulfonate groups (–SO_3_Na) from oxygen-rich DR 23 at the resin surface [77], and the trace chlorine identified comes from the synthesis or purification process of the dye. Since X4 is hydrophobic, it attracts the aromatic moieties of the dye. Although DR 23 has hydrophilic sulfonate groups, the affinity of its naphthalene nucleus for the styrene matrix of the resin allows for its fixation [70]. The appearance of Na (0.5 wt%) and Cl (0.1 wt%), which are absent in the untreated sample, confirms the presence of DR 23 in the form of disodium salt on the resin surface. Although sulfur (5.1 wt %) from the composition of the dye is not detected in the adsorbed sample, this is explainable by the low concentration of the dye and the limitations of the EDS technique. Overall, the observed changes validate the adsorption of DR 23 on X4, reinforcing its applicability in heavy metal recovery paths [78], chelating matrix developments [73,79], and eco-friendly hybrid water purification processes [74,80].

### 3.6. TG/DSC Analysis of Solid Phases

**Figure 11:**
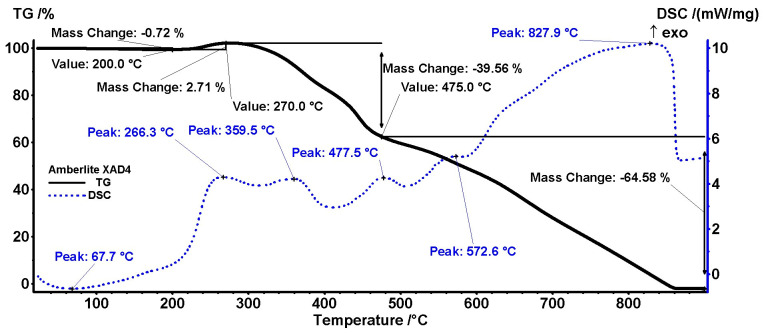
TG/DSC thermogram of X4.

The X4 sample is stable up to 200 °C, with the small mass loss of 0.72% being most probably due to residual humidity [81]. After 200 °C, the sample gains mass, 2.71%, the process being accompanied by an exothermic effect at 266.3 °C, indicating an oxidation reaction (type of oxidation of the double bond with introduction of –OH or oxidation of C–OH with transformation into COOH, and therefore with mass increase). A similar mass increase was reported previously for XAD4 resins [13]. The complex degradation process starts after 270 °C, with oxidation and fragmentation reactions overlapped as indicated by the sinusoidal DSC curve [82]. After 475 °C, the carbonaceous residual mass is slowly oxidized and eliminated up to 860 °C [83] (Figure 11).

**Figure 12:**
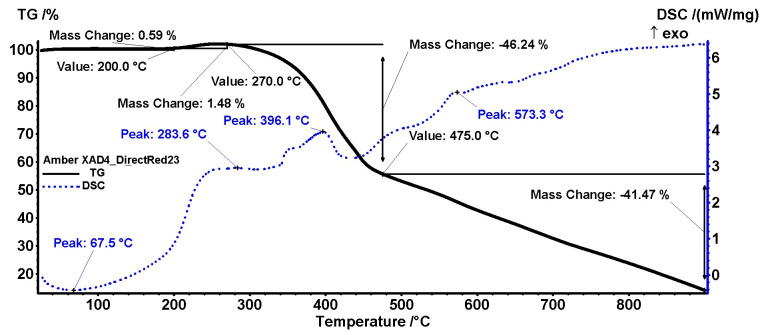
TG/DSC thermogram of X4 loaded with DR 23.

The X4 sample with DR 23 has a similar thermal behavior as pristine resin up to 380 °C, minor differences being observed in the position of thermal effects. After 380 °C, the sample containing dye DR 23 exhibits a larger mass loss up to 660 °C due to the degradation of the adsorbed organics [84]. As the DR 23 has an inorganic residual part that cannot be burned away, after 660 °C, the mass loss of the loaded resin becomes smaller than that of the pristine resin, the residual mass allowing for an estimation of the DR 23 load to 31.45% [85] (Figure 12).

**Figure 13:**
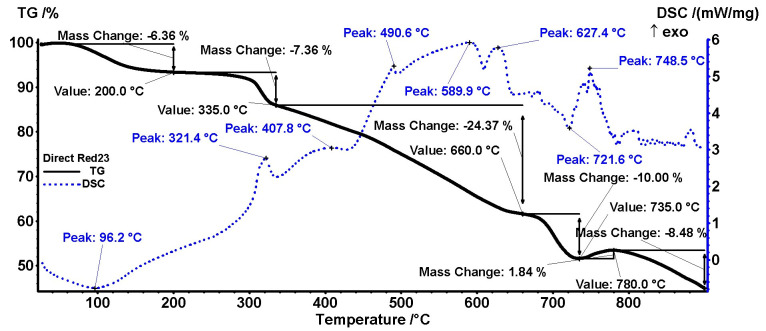
TG/DSC thermogram of DR 23.

The DR 23 (C_35_H_25_N_7_Na_2_O_10_S_2_), 813.7 g/mol) loses 6.36% of its mass up to 200 °C, the process being accompanied by an endothermic effect with minimum at 96.2 °C, suggesting a possible dehydration process. The second mass loss event, 7.36%, starts slowly after 200 °C, is more obvious after 250 °C, and is associated with an exothermic effect at 321.4 °C, indicating a partial oxidation of the dye molecule [86,87]. Between 335 and 660 °C, the sample loses 24.37% of its mass, continuously, in an oxidative-degradative process, in which exothermic effects are dominant, with peaks at 490.6, 589.9 and 627.4 °C, a common feature for aromatic azo dyes [88,89]. Finally, the sample exhibits a complex behavior after 660 °C, with decomposition of inorganic salts, followed by oxidation and again decomposition [90] (Figure 13).

## 4. Discussion

The adsorption behavior of DR 23 on X4 was first evaluated through kinetic, equilibrium, and solid-phase characterization analyses.

The kinetic results revealed a multistage adsorption process, with an initial rapid uptake followed by a slower diffusion-controlled phase. The pseudo-second-order model provided the best correlation (R^2^ = 0.9550), indicating that the overall rate is influenced by both surface interactions and diffusion limitations. The progressive decrease in the adsorption rate suggests the sequential occupation of high-energy sites, followed by slower adsorption on less accessible regions of the resin.

Equilibrium data fitted well to the Langmuir isotherm, confirming monolayer adsorption on homogeneous sites. This behavior indicates a predictable and saturable removal mechanism, allowing for reliable estimation of the resin dose required for reducing dye concentrations to environmentally relevant levels. The maximum adsorption capacity (~56 mg/g) demonstrates that X4 can efficiently retain DR 23 even at elevated pollutant loads, which is particularly relevant for industrial effluents.

Spectroscopic and microscopic analyses further support the formation of stable dye–resin associations. FTIR spectra showed shifts in the –N=N– and –SO_3_^−^ functional groups after adsorption, indicating specific interactions between DR 23 and the polymeric matrix. SEM/EDX analysis confirmed the presence of sulfur and nitrogen from the dye on the resin surface, demonstrating effective sequestration. Thermal analysis (TG/DSC) showed that the structural integrity of X4 is preserved after adsorption, indicating that the process does not compromise the stability of the material.

From a toxicological perspective, the efficient and stable removal of DR 23 is significant. Azo dyes such as DR 23 can inhibit photosynthesis, disrupt microbial communities, and generate mutagenic aromatic amines under anaerobic conditions [91]. By reducing the dissolved concentration of the dye, the adsorption process directly lowers ecological exposure and mitigates the formation of hazardous metabolites.

Based on the combined kinetic, isotherm, and characterization results, a mechanistic interpretation can be proposed. The adsorption of DR 23 onto X4 involves an initial film diffusion step, followed by intraparticle diffusion and final pore saturation. The strong affinity observed experimentally is consistent with π–π interactions between the aromatic rings of DR 23 and the styrene–divinylbenzene backbone of X4, contributing to the stable immobilization of the dye within the resin matrix. This stable binding reduces the likelihood of desorption under environmental conditions, supporting the applicability of X4 in wastewater treatment strategies aimed at minimizing toxicological risks associated with persistent azo dyes.

## 5. Conclusions

This study provides a comprehensive evaluation of X4 as an effective adsorbent for the removal of DR 23 from aqueous media. The adsorption behavior, assessed through equilibrium and kinetic analyses, confirms that X4 exhibits strong affinity toward the dye molecules, driven predominantly by surface interactions and π–π or hydrophobic forces inherent to the resin’s polymeric matrix.

The equilibrium data conforms well to widely applied isotherm models, indicating the presence of energetically favorable adsorption sites and a high degree of surface heterogeneity.

Kinetic modeling further reveals that the adsorption process proceeds rapidly and is best described by physisorption-controlled mechanisms, underscoring the resin’s suitability for practical treatment scenarios requiring fast pollutant removal. Analysis of the presented data confirms that X4 (a styrene–divinylbenzene polymer matrix) functions as a high-performance hydrophobic adsorbent for the anionic DR 23.

The efficiency of X4 is based on π–π interactions between the aromatic rings of the resin and the naphthalene/azo structure of the DR 23, along with Van der Waals forces and hydrophobic attractions. The non-polar matrix is optimal for large organic molecules, such as direct dyes (DR 23). Compliance with Freundlich or Langmuir isotherm models suggests a surface with heterogeneously distributed active sites, allowing for high loading even at low residual concentrations.

The fact that the process is fast and controlled by physisorption (pseudo second-order model) indicates a low energy barrier for molecule attachment, which is ideal for continuous industrial flows. Due to the nature of the mechanism (physisorption), regeneration is much easier than in the case of chemisorption.

As a result of DR 23 having sulfonic (anionic) groups, increasing the pH above the zero-charge point of the resin can create electrostatic repulsions, facilitating the release of the dye.

Thus, X4 is distinguished both by its high adsorption capacity (not the highest compared to nanomaterials), but especially by its operational robustness and ease of reuse in numerous adsorption–desorption cycles.

Collectively, these findings establish X4 as a technically viable and environmentally relevant adsorbent for dye-laden wastewater, offering advantages such as high removal efficiency, structural stability, and potential for regeneration.

The FTIR spectrum of the unloaded resin displayed characteristic bands associated with the aromatic polymeric matrix, including C–H stretching vibrations, aromatic C=C skeletal modes, and C–O functionalities typical of the styrene–divinylbenzene backbone. Following adsorption, noticeable shifts in peak positions and changes in band intensities were observed, particularly in regions corresponding to aromatic ring vibrations and functional groups associated with the dye molecule. These spectral modifications indicate the establishment of intermolecular interactions between the dye and the resin surface. The appearance of or enhancement in bands associated with –N=N– azo groups and sulfonate functionalities in the dye-loaded resin further confirms the successful immobilization of DR 23 onto X4. The observed spectral changes suggest that adsorption is primarily governed by π–π interactions between the aromatic structures of the dye and the resin, as well as possible hydrogen bonding.

SEM/EDAX analysis confirms the structural changes in X4 following the adsorption process of the DR 23. The low-magnification images, 50×, highlight that the resin is presented in the form of polydisperse spherical beads, with dimensions ranging between 440 µm and 750 µm.

The integrity of the spheres after adsorption demonstrates the high mechanical stability of the styrene–divinylbenzene support. At intermediate magnifications (4000×–5000×), a heterogeneous surface is observed. The areas of microporosity of the resin appear partially obstructed by dye deposits. The presence of “islands of material” indicates a high affinity of DR 23 for the polymer matrix, the adsorption process being visible through the formation of crusts on the surface of the pores. Analysis of the structure at extreme magnification (40,000×) shows a nodular texture presented by high-resolution micrography (3 µm).

This morphology confirms the formation of a dense dye layer flooding the surface, suggesting an adsorption mechanism that tends towards saturation of the external active sites, an aspect correlated with the decrease in carbon (from 95.8% to 90.1%). The increase in oxygen (8.8%) and the appearance of Na (0.5%) represent the chemical fingerprint of the DR 23. The presence of Al (0.5%) can be attributed to residual impurities or to the signal from the sample support during analysis. The correlation of the data indicates that the adsorption of DR 23 on X4 is an efficient process, facilitated by the macroreticular structure of the resin.

The TG/DSC analysis confirmed the high thermal stability of X4 and provided clear evidence of structural changes following DR 23 adsorption. The unloaded resin exhibited the expected multi-stage degradation profile characteristic of styrene–divinylbenzene polymers, with minimal initial mass loss attributed to physically adsorbed moisture. After dye loading, slight shifts in decomposition temperatures and modified enthalpic events were observed, indicating the incorporation of dye molecules within the polymer matrix and their influence on the resin’s thermal behavior. Overall, the thermal analysis verifies that adsorption does not compromise the structural integrity of X4 and supports the formation of stable dye–resin interactions.

This study contributes valuable insights into the adsorption mechanisms governing dye–resin interactions and provides a scientific foundation for the integration of X4 into industrial wastewater treatment frameworks, particularly within the textile and dye manufacturing sectors, where synthetic dyes pose persistent ecological risks.

## Figures and Tables

**Table 1:** Morris Weber, Elovich, PFO and PSO kinetics parameters.

Morris Weber (intraparticle diffusion)	$q_{t}=k_{id}{\left(t\right)}^{0.5}$	(4)	k_id_ (mg/g·h^−0.5^)	C (mg/g)	R^2^
			7.91	1.11	0.9607
Elovich	$q_{t}=\ln(\alpha \beta )+\frac{1}{\beta }\mathrm{lnt}$	(5)	α (mg/g·h)	β (g/mg)	R^2^
			0.002	0.0711	0.9550
PFO	$\log\left(q_{e}-q_{t}\right)=\log q_{e}-\left(\frac{k_{1}}{2303}\right)t$	(6)	k_1_ (g/mg·h)	Q_e_ (mg/g)	R^2^
			0.11	85.07	0.8970
PSO	$\frac{t}{q_{t}}=\frac{1}{k_{2}q_{e}2}+\frac{t}{q_{e}}$	(7)	k_2_ (g/mg·h)	Q_e_ (mg/g)	R^2^
			0.001	73	0.9648

**Table 2:** Langmuir, Freundlich, Temkin, and Dubinin–Radushkevich isotherm parameters.

Langmuir		Temkin–Phyzev	
Q_o_ (mg/g)	56.8	A (L/mg)	14.1
b (L/mg)	0.58	b_T_ (J/mol)	288
R_L_	0.01	B	8.45
R^2^	0.9990	R^2^	0.8853
**Freundlich**		**Dubinin–Radushkevich**	
K_f_ (mg/g)	13.6	q_m_ (mg/g)	47.1
1/n	0.28	β (mol^2^/kJ^2^)	2 × 10^−7^
n	3.63	E (kJ/mol)	1.58
R^2^	0.7675	R^2^	0.8931

## Data Availability

The original contributions presented in this study are included in this article. Further inquiries can be directed to the corresponding author.
